# Initial Care Pathway in Acute Heart Failure From Home to Hospital

**DOI:** 10.1002/clc.70161

**Published:** 2025-06-11

**Authors:** Pia Harjola, Veli‐Pekka Harjola, Òscar Miró, Said Laribi, Tuukka Tarvasmäki

**Affiliations:** ^1^ Emergency Medicine University of Helsinki and Department of Emergency Medicine and Services, Helsinki University Hospital Helsinki Finland; ^2^ Emergency Department, Hospital Clínic University of Barcelona Catalonia Spain; ^3^ School of Medicine and CHU Tours, Emergency Medicine Department Tours University Tours France; ^4^ Cardiology University of Helsinki and Heart and Lung Center, Helsinki University Hospital Helsinki Finland

**Keywords:** acute heart failure, emergency medical services, management, outcomes, pre‐hospital

## Abstract

**Introduction:**

The prognosis of acute heart failure (AHF) remains poor. Studies focusing on the time‐sensitivity of early AHF management have reported controversial results. Thus, our aim is to review current studies focusing on AHF patients using emergency medical services (EMS), their early management, and patient outcomes.

**Methods:**

We searched the recent literature in PubMed and Scopus for studies comparing AHF patients arriving at the hospital by EMS to those self‐presenting (non‐EMS) at ED (emergency department) from database inception until November 2022.

**Results:**

The literature search found five studies fulfilling our inclusion criteria. The percentage of AHF patients using EMS varied in these studies: 11.5% (100/873) in Finnish FINN‐AKVA II, 22.1% (236/1068) in Canadian ASCEND‐HF, 35.5% (5129/14454) in a Pakistan Heart Failure‐registry study, 52.8% (3224/6106) in Spanish SEMICA, and 61.8% (309/500) in the European EURODEM study. The pre‐hospital management differed across the reviewed studies. The use of NIV was rare, ranging from zero to four percent. Vasodilators and diuretics were more commonly used. Although, the differences in the use were obvious (range from 7.1% to 22.0%, and 0.0% to 29.0% accordingly). Three of the studies reported significantly higher 30‐day mortality among EMS patients compared to non‐EMS patients: ranging from 5.6% versus 3.5%, *p* < 0.001% to 15.0% versus 6.9%, *p* < 0.001.

**Conclusion:**

The use of EMS, as well as pre‐hospital management, varies between the international cohorts and registries. The pre‐hospital AHF management is generally limited. Moreover, EMS patients tend to have worse outcomes compared to non‐EMS patients.

## Graphical abstract

The use of EMS among AHF patients, their early management, and patient outcomes.

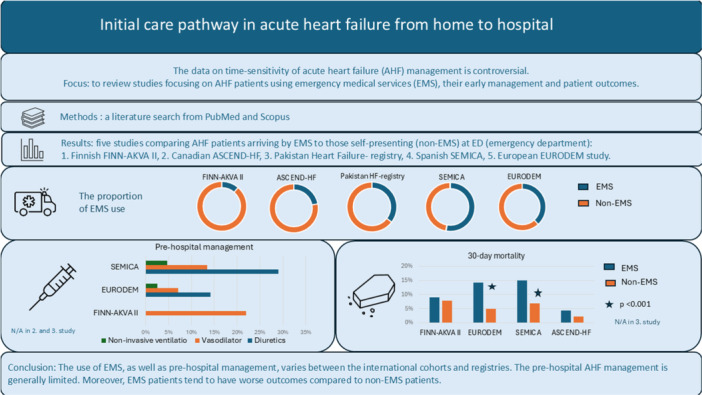

**Abbreviations**
AHF: acute heart failure
ALS: advanced life support
BLS: basic life support
CKD: chronic kidney disease
COPD: chronic obstructive pulmonary disease
ED: emergency department
EMS: emergency medical services
HR: heart rate
IQR: inter quartile range
IV: intravenous
LOS: length of hospital stay
NIV: noninvasive ventilation
RR: respiratory rate
SBP: systolic blood pressure
SD: standard deviation
SpO2: peripheral oxygen saturation

## 1 Introduction

Heart failure remains one of the leading causes of mortality and morbidity worldwide. Acute heart failure (AHF) can present either as sudden worsening or new onset of signs and symptoms of heart failure. Both AHF presentations require rapid evaluation and management [1]. The initial AHF management is provided by emergency departments (ED) and emergency medical services (EMS) [2, 3]. The EMS use varies from 25% to 62% among AHF patients admitted to ED [4, 5, 6, 7].

Pre‐hospital management protocols for AHF exist, but the treatment options differ between different levels of EMS units and regions [8, 9]. Traditionally, EMS units are divided into two levels based on the personnel and management options available [10]. Advanced life support (ALS) units are staffed by paramedic or emergency physician and contain invasive managements (e.g., intravenous (IV) diuretics, IV vasodilators, mechanical ventilation) and noninvasive ventilation (NIV). Basic life support (BLS) units provide patient monitoring and are staffed with paramedics or emergency technicians.

Lately, the European Society of Cardiology guidelines have highlighted the role of pre‐hospital AHF management [1, 11, 12]. However, clinical trials examining the time sensitivity of AHF management have reported controversial results [13, 14, 15, 16, 17, 18, 19, 20]. Here we review studies focusing on AHF patients using EMS, their early management, and outcomes.

## 2 Materials and Methods

We conducted a literature search from PubMed and Scopus to find published articles comparing AHF patients using EMS and those self‐presenting at ED (non‐EMS) from database inception until November 2022. The two databases were selected to cover a broad range of research areas. The literature search included keywords “acute heart failure”, “AHF”, “heart failure (HF)”, “HF”, “emergency medical services”, “EMS”, “pre‐hospital”, “prehospital” and “ambulance”, which were thought to cover the scope of this review. Titles and abstracts were reviewed for the first round of literature screening. Based on the relevance of abstracts and titles, full‐text articles were further reviewed. Full‐text articles fulfilling the inclusion criteria were evaluated and compared to find similarities between the different EMS populations, initial managements, and patient outcomes.

For two of the studies, the FINN‐AKVA II and the EURODEM, data were available for further analyses. For the FINN‐AKVA II, the 30‐day mortality and the ED management were analysed with between‐group comparisons by a *χ* ^2^. Furthermore, independent predictors of 1‐year mortality were analysed with multivariable logistic regression. The variables were initially selected based on clinical relevance and previous literature [21, 22, 23, 24]. The final variable selection was done with forward and backward logistic regression from the following variables: age, gender, ED arrival mode (i.e., EMS), systolic blood pressure (SBP), heart rate (HR), respiratory rate (RR), peripheral oxygen saturation (SpO2), sodium, potassium, haemoglobin, confusion, a history of chronic obstructive pulmonary disease (COPD), chronic kidney disease (CKD), and cognitive dysfunction/dementia. *p* value < 0.05 was used for variable inclusion and > 0.1 for elimination. For the EURODEM, we analysed the use the pre‐hospital managements. The additional analysis was made with SPSS 26. The results are reported as medians with interquartile range (IQR) and mean with standard deviation (SD) for continuous variables, and numbers (*n*) with percentages for nominal variables.

## 3 Results

**Figure 1:**
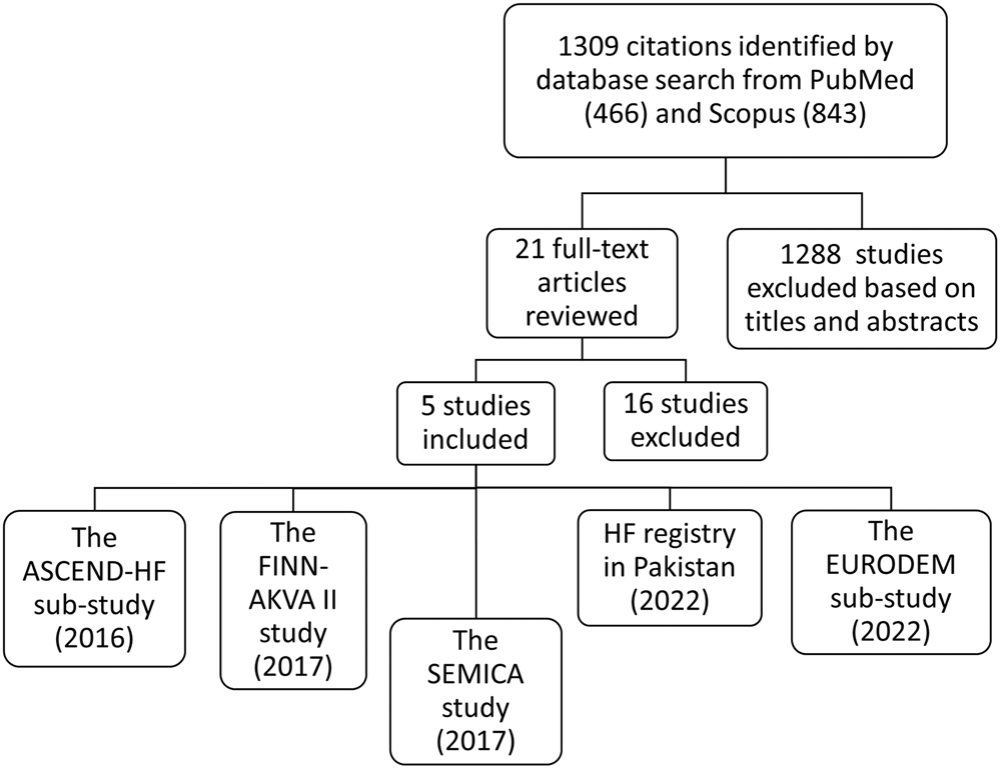
A summary of the literature search.

We found a total of 1309 published articles. Most of the citations were excluded already during the first step, as the used keywords also corresponded to other research topics such as ST‐elevation myocardial infarction. Finally, five studies focused on the differences between AHF patients using EMS and non‐EMS patients fulfilling the inclusion criteria (Figure 1). The included studies were a sub‐study of the Canadian ASCEND‐HF published in 2016 [25], a Finnish FINN‐AKVA II study [26], a Spanish SEMICA study [27] published in 2017, a sub‐study of the European EURODEM study [28], and a Pakistan Heart Failure‐registry study [29] published in 2022.

The proportion of AHF patients arriving at the ED by EMS varied greatly between the studies (Table 1). The FINN‐AKVA II and the SEMICA reported the level of the EMS unit used. In both studies, more than half of AHF patients were transported by lower‐level EMS units, 57% of patients in the FINN‐AKVA II and 53% in the SEMICA.

**Table 1:** Patient characteristics.

AHF‐study: Country (data collection year)	FINN‐AKVA II [26] (*N* = 873) Finland (2012–2013)		EURODEM [28] (*N* = 500) Europe (2014)		SEMICA [27] (*N* = 6106) Spain (2011–2014)		ASCEND‐HF [25] (*N* = 1068) Canada (2009–2010)		Pakistan Heart Failure‐registry study [29] (*N* = 14 454) (2019–2020)	
	EMS	non‐EMS	EMS	non‐EMS	EMS	non‐EMS	EMS	non‐EMS	EMS	non‐EMS
	11.5%	88.5%	61.8%	38.2%	52.8%	47.2%	22.1%	77.9%	35.5%	64.5%
Age, years	78 (70–83)	78 (69–84)	80 (71–85)	75 (65–81)*	64.5% > 80 years	50.6% > 80 years*	72 (61–81)	65 (55–77)*	66 (SD 8.0)	61 (SD 7.3)**
Female gender	55.0%	47.6%	56.4%	42.1%**	59.5%	53.2%*	44%	33%	37%	28%**
Comorbidities										
Previous heart failure	74.0%	63.9%***	61.6%	59.8%	62.4%	55.2%*	48%^a^	45%^a^	71.2%^a^	66.7%^a^^,^ *
Ischemic heart disease	50.0%	36.9%***	41.1%	42.5%	31.1%	29.5%	61%	60%	53.2%	54.1%
Hypertension	68.0%	63.2%	73.8%	66.5%	85.8%	83.4%***	88%	81%***	24.8%	26.7%
Atrial fibrillation	47.0%	43.6%	36.3%	34.8%	47.6%	49.8%	38%	43%	NA	NA
CKD	38.0%	17.1%*	27.4%	21.7%	27.1%	23.5%**	NA	NA	22.1	17.7***
COPD	20.0%	15.0%	28.8%	25.8%	27.9%	23.9%*	29%	26%	8.3%	8.9%
Dementia	3.0%	9.2%***	18.7%	7.2%**	16.7%	9.7%*	NA	NA	NA	NA

*Note:* The continuous values are reported as median with interquartile range, median (IQR), and nominal values as percentages.

Abbreviations: CKD = chronic kidney disease, COPD = chronic obstructive pulmonary diseases, ED = emergency department, EMS = emergency medical services, HF = heart failure, NA= not available.

*p* values are comparisons between EMS and non‐EMS patients inside the studies.

^a^ Hospitalized for heart failure during the prior year.

* *p* < 0.001

** *p* < 0.01

*** *p* < 0.05.

Table 1 shows patient characteristics. Previous heart failure, hypertension, atrial fibrillation, and ischemic heart disease were the most common comorbidities among all AHF patients of the five studies. Although there were differences between the studies, older age, hypertension, previous heart failure, CKD, and dementia tended to be more common among EMS patients compared to non‐EMS patients.

The use of pre‐hospital medications is shown in Table 2. In the FINN‐AKVA II, the administered pre‐hospital medications were vasodilators and IV opiates. In the EURODEM and the SEMICA, IV diuretics were the most frequently used medications, followed by vasodilators. In the FINN AKVA II, none of the patients received NIV, whereas in the SEMICA 4.8% of EMS patients and in EURODEM 2.6% of EMS patients received NIV.

**Table 2:** Pre‐hospital acute heart failure management.

Pre‐hospital management	FINN‐AKVA II (*N* = 873)	EURODEM (*N* = 500)	SEMICA (*N* = 6106)	ASCEND‐HF (*N* = 1068)	Pakistan HF registry‐study (*N* = 14 454)
IV/PO diuretics	0.0%	14.2%	29.0%	NA	NA
Sublingual/IV vasodilator	22.0%	7.1%	13.5%	NA	NA
IV opiate	21.0%	NA	NA	NA	NA
Noninvasive ventilation	0.0%	2.6%	4.7%	NA	NA

Abbreviations: HF = heart failure, IV = intravenous, NA= not available, PO = per os.

*p* values are comparison between EMS and non‐EMS patients inside the studies.

FINN‐AKVA II, the EURODEM, and the Pakistan HF‐registry study reported vital signs on ED admission (Table 3). EMS patients tended to have higher RR and HR, and lower SpO_2_ compared to non‐EMS patients. Furthermore, in the EURODEM, EMS patients had the highest RR compared to EMS patients in the other three studies.

**Table 3:** Vital signs on admission to the emergency department.

	FINN‐AKVA II (*N *= 873)		EURODEM (*N* = 500)		SEMICA (*N* = 6106)		ASCEND‐HF sub‐study (*N* = 1068)		Pakistan HF registry‐study (*N* = 14 454)	
Vital signs	EMS	non‐EMS	EMS	non‐EMS	EMS	non‐EMS	EMS	non‐EMS	EMS	non‐EMS
SBP (mmHg)	137 (117–159)	130 (108–150)	140 (120–156)	143 (122–162)	NA	NA	130 (116–140)	124 (110–40)**	110 (87–30)	110 (89–132)
DBP (mmHg)	81 (70–97)	83 (69–101)	80 (66–92)	80 (67–90)	NA	NA	72 (64–84)	74 (65–85)	70 (65–83)	71 (65–83)
Heart rate (bpm)	82 (70–100)	80 (69–97)	90 (75–110)	85 (75–104)	NA	NA	78 (69–90)	79 (70–90)	94 (75–125)	93 (74–120)***
Respiratory rate (per min)	21 (18–26)	20 (18–26)	24 (19–30)	21 (18–26)***	NA	NA	22 (20–24)	222 (20–24)	20 (17–24)	20 (17–24)***
SpO_2_ (%)	94 (90–96)	95 (91–97)	94 (90–97)^a^	94 (89–96)^a^	NA	NA	NA	NA	82 (SD 11)	87 (SD 9)***

*Note:* The continuous values are reported as median with interquartile range, median (IQR), and nominal values as percentages.

Abbreviations: BMP = beats per minute, DBP = diastolic blood pressure, HF = heart failure, SBP = systolic blood pressure, SpO_2_ = peripheral oxygen saturation, NA = not available.

*p* values are comparisons between EMS and non‐EMS patients inside the studies.

^a^ SpO_2_ with supplementary oxygen.

** *p* < 0.01

*** *p* < 0.05.

The SEMICA and the EURODEM reported the number of patients admitted to a ward (Table 4). In both studies, EMS patients were admitted significantly more often and had significantly higher in‐hospital mortality. The SEMICA, the EURODEM, and the Pakistan HF‐registry study, also reported a significantly higher 30‐day mortality among EMS patients compared to non‐EMS patients (Table 4). In the ASCEND‐HF and Pakistan Heart Failure‐registry study, the 180‐day mortality was significantly higher among EMS patients. In the EURODEM, EMS was an independent predictor of 30‐day mortality (OR 2.96, 95% CI 1.27–6.92, *p* = 0.012). Other variables associated with 30‐day mortality in the EURODEM included male gender (OR = 2.75, 95% CI 1.32–5.76, *p* = 0.007), confusion (OR = 5.28, 95% CI 2.30–12.16, *p* < 0.001), SpO_2_ (OR = 0.94, 95% CI 0.90–0.98, *p* = 0.008), sodium level (OR = 0.91, 95% CI 0.86–0.96, *p* = 0.002), and haemoglobin (OR = 0.80, 95% CI 0.69–0.92, *p* = 0.002). In the FINN‐AKVA II, no independent predictors of mortality were identified in logistic regression analysis. In the ASCEND‐HF, EMS arrival tended to increase the risk of 30‐day mortality (OR 2.12, 95% CI 0.94–4.79). In the Pakistan Heart Failure‐registry study, the length of hospital stay (Table 4), and 30‐day rehospitalization rates were significantly higher among EMS patients compared to non‐EMS (56.3% vs. 34.5%, *p* < 0.001).

**Table 4:** Patient outcomes.

	FINN‐AKVA II (*N* = 873)		EURODEM (*N* = 500)		SEMICA (*N* = 6106)		ASCEND‐HF sub‐study (*N* = 1068)		Pakistan HF registry‐study (*N* = 14454)	
	EMS	non‐EMS	EMS	non‐EMS	EMS	non‐EMS	EMS	non‐EMS	EMS	non‐EMS
Admission to a ward	NA	NA	62.5%	51.8%***	88.0	69.8%*	NA	NA	NA	NA
Admission to intensive care unit	NA	NA	15.3%	11.0%	4.1	0.9%*	NA	NA	NA	NA
Length of hospital stay	6 (3‐10)	7 (3‐11)	7 (3‐12)	7 (1‐11)	NA	NA	NA	NA	14 (SD 6.2)	8 (SD 4.1)**
In‐hospital mortality	6.0%	7.1%	8.7%	3.1%***	12.5%	7.0%*	NA	NA	6.8%	5.2%
30‐day mortality	9.0%	7.8%	14.3%	4.9%*	15.0%	6.9%*	4.3%	2.2%	5.6%	3.5%*

Abbreviations: EMS = emergency medical services, HF = heart failure, NA = not available.

In SEMICA the *p* value was calculated using the linear trend *χ* ^2^ test.

*p* values are comparisons between EMS and non‐EMS patients inside the studies.

* *p* < 0.001

** *p* < 0.01

*** *p* < 0.05.

In the SEMICA, independent factors associated with EMS use were analysed. These factors included age over 80 years (OR 1.5, 95% CI 1.3–1.74, *p* < 0.001), female sex (OR 1.27, 95% CI 1.10–1.47, *p* < 0.001), Barthel index < 60 (OR 1.95, 95% CI 1.56–2.45, *p* < 0.001), NYHA class III‐IV (OR 1.22, 95% CI 1.02–1.45, *p* < 0.05), COPD (OR 1.49, 95% CI 1.27–1.75, *p* < 0.001), cold skin (OR 1.89, 95% CI 1.44–2.48, *p* < 0.001), and a previous episodes of AHF (OR 1.20, 95% CI 1.04–1.39, *p* < 0.01).

## 4 Discussion

In this paper, we aimed to illustrate the AHF population using EMS, their early management, and outcomes, and compare them to AHF patients self‐presenting at the ED. The current literature comparing these two patient groups is scarce. Firstly, the reviewed studies showed that EMS use and pre‐hospital management of AHF patients varied between the countries. Furthermore, AHF patients using EMS tended to be more often females, and had more comorbidities compared to non‐EMS patients. Finally, EMS patients had mostly worse short‐term outcomes compared to non‐EMS patients.

This review showed that the use of EMS differs between countries, as could be predicted. Longer transportation distances have been demonstrated to be linked to higher EMS use, which was also seen in the reviewed studies [30]. In the FINN‐AKVA II, including patients from the relatively small Helsinki metropolitan area, the EMS use was the lowest, whereas in the EURODEM study, including larger European cities, the use of EMS was more frequent. Furthermore, the EMS referral criteria vary between countries [30, 31]. Thus, the geographical differences and local EMS policies partially explain the varying frequencies of EMS use, in addition to patients' decision making.

In the reviewed studies, EMS patients tended to be more often older females. In addition, factors associated with frailty, for example, dementia seemed to, in part, affect the EMS use. Among comorbidities, hypertension, CKD, previous heart failure, ischemic heart disease, and dementia were more common among EMS patients compared to non‐EMS patients. However, there were some differences in the distribution between the studies. In contrast, patients self‐presenting at the ED, that is, non‐EMS patients, tended to have fewer comorbidities and factors associated with frailty, indicating higher functional ability.

On arrival to ED, EMS and non‐EMS patients' illness severity did not differ greatly. Yet, we did not have the initial vital signs of EMS patients measured in the pre‐hospital setting and thus it must be considered that EMS patients vital sign might have improved during the transportation. Overall, it seemed that lower reserves to tolerate AHF due to comorbidities and older age tended to promote EMS use more than illness severity.

The use of pre‐hospital AHF management was both low and variable. The limited use of pre‐hospital medications has been addressed earlier as well [32]. Difficulty to diagnose AHF, especially in the pre‐hospital setting, has been shown to impact the implementation of AHF managements [33, 34]. Additionally, adverse effects have been reported when AHF managements are administered to misdiagnosed patients in EMS [10, 35]. Furthermore, local EMS practices differ, and the equipment and management capabilities vary between different levels of EMS units and EMS regions [8, 9]. Therefore, EMS units in Finland, not containing IV diuretics, provided vasodilators and IV opiates, whereas EMS units in other parts of Europe mainly used diuretics. Moreover, fewer than half of AHF patients were managed by ALS units equipped with IV management capabilities.

Finally, in four of the reviewed studies, EMS patients had significantly worse short‐term outcomes compared to non‐EMS patients, which has also been observed among chest pain patients [30, 36, 37]. The EURODEM was the only study to report all the independent factors associated with the worst outcomes. These factors included gender and clinical findings (confusion, as well as lower SpO_2_, sodium, and haemoglobin). However, no comorbidities were reported to be independently associated with the outcome. Furthermore, EMS was reported as an independent predictor of mortality, consistent with the prior studies of AHF and chest pain patients [30, 36]. However, the EMS patients were significantly older compared to non‐EMS patients and had, in two of the studies, significantly more often dementia, factors independently associated with higher mortality [38, 39, 40, 41, 42, 43]. In addition, in the SEMICA, EMS use was reported to be related to frailty, another factor associated with worse outcomes [44, 45].

Some limitations must be addressed. We found only five studies comparing EMS and non‐EMS patients suffering from AHF. The EMS regions and populations cannot be compared equally, as the EMS setting varies between countries [8, 9]. Also, the use of different levels of EMS units was not reported in all the studies. Most likely, the severity of AHF would affect the use of EMS. Unfortunately, none of the studies had categorized the patients according to the severity of AHF or reported the clinical classes. In addition, some of the parameters were only reported in two of the studies, and therefore, the assumptions should be evaluated with caution. However, we feel that this study brings more knowledge about the AHF patients using EMS and their outcomes.

## 5 Conclusion

The data focusing on EMS and non‐EMS patients is limited. The use of EMS and pre‐hospital managements vary between countries. The patient populations are heterogenic, and patients' age, comorbidities, and frailty seem to affect EMS use more than illness severity. EMS patients have worse outcomes, which seems to be influenced by their older age and frailty. Overall, this review shows that the use of EMS does not improve patient outcomes, but is associated with a worse prognosis. Future studies focusing on the independent factors associated with EMS use and the differences between EMS and non‐EMS patients should be carried out specially to find out the reason for the EMS patients' worse outcomes. In addition, the pre‐hospital management is variable and thus future randomized controlled trials are needed to determine the prognostic effect of pre‐hospital AHF managements, for example, the use of intravenous furosemide, the mainstay of AHF treatment, to form a proper understanding of the role of pre‐hospital management in AHF. Also, oxygen supplementation and ventilatory support could be studied in carefully selected patient populations with specified selection criteria.

## Ethics Statement

Additional data analyses were made for FINN‐AKVA II and EURODEM. The studies were performed in accordance with the Declaration of Helsinki. For EURODEM the ethics committee approvals were obtained for different regions depending on the local requirements.

## Consent

Patient consent for data collection was included if requested by the ethics committee; in most participating sites, it was verbal.

## Conflicts of Interest

The authors declare no conflicts of interest.

## Data Availability

Additional analyses were made for FINN‐AKVA II and EURODEM. The FINN‐AKVA II and EURODEM databases are not publicly available. The data sets used and/or analyzed during the current study are available from the corresponding author on reasonable request.
